# Aspiration therapy for obesity; a safe and effective treatment

**DOI:** 10.1186/s40608-016-0134-0

**Published:** 2016-12-28

**Authors:** Erik Norén, Henrik Forssell

**Affiliations:** 1 Department of Surgery, Blekinge County Hospital, Lasarettsvägen, Karlskrona, S-371 85 Sweden; 2 Blekinge Center of Competence, Blekinge County Council, Karlskrona, Sweden

**Keywords:** Obesity treatment, Gastrostomy, Aspiration therapy

## Abstract

**Background:**

This study evaluated the efficacy and safety of the novel AspireAssist® Aspiration Therapy System for treatment of obesity, and its effect on patient’s quality of life.

**Methods:**

A prospective observational study with 25 obese subjects, mean age 48 years (range 33–65), was performed. A custom gastrostomy tube (A-tube, Aspire Bariatrics) was percutaneously inserted during a gastroscopy performed under conscious sedation. Drainage and irrigation of the stomach were performed 3 times daily, 20 min after each meal, for 1–2 years. Efficient aspiration required thorough chewing of ingested food. Treatment included a cognitive behavioral weight loss program.

**Results:**

Mean body mass index (BMI) at inclusion was 39.8 kg/m^2^ (range 35–49). After 1 year mean (SD) BMI was 32.1 kg/m^2^ (5.4), *p* < 0.01, and excess weight loss was 54.4% (28.8), *p* < 0.01. Quality of life, as measured with EQ-5D, improved from 0.73 (0.27) to 0.88 (0.13), *p* < 0.01. After 2 years BMI was 31.0 kg/m^2^ (5.1), *p* < 0.01, and excess weight loss was 61.5% (28.5), *p* < 0.01. There were no serious adverse events or electrolyte disorders. Compliance was 80% after 1 year and 60% after 2 years.

**Conclusions:**

Aspiration therapy is an efficient and safe treatment for obesity, and weight reduction improves quality of life. Excess weight was approximately halved in a year, with weight stability if treatment was continued.

**Trial registration:**

Trial Register ISRCTN 49958132. Retrospectively registered 28/02/2014.

## Background

Obesity, as defined by World Health Organization as body mass index (BMI) greater than 30 kg/m2, is a major global health problem with increasing prevalence. Obesity has more than doubled worldwide since 1980. In 2014, more than 1.9 billion adults, of 18 years and older, were overweight. Most of the world’s population live in countries where overweight and obesity cause higher mortality than underweight [1]. In young and middle age all-cause mortality risk appears to be directly related to increase in BMI [2]. There is a well-established association between obesity and decreased quality of life [3] as well as morbidity from diabetes mellitus type 2, elevated blood pressure, dyslipidemia and cardiovascular complications as coronary artery disease and cerebrovascular insult [4]. It has recently been shown that obesity also is an avoidable cause of some specific forms of cancer [5].

Alarmingly, it has been shown that obese mothers affect the phenotype of their offspring, rendering the child more susceptible to obesity. This effect is irreversible but preventable by bariatric surgery prior to pregnancy [6, 7]. On the other hand, bariatric surgery before pregnancy increases risk of preterm and small for gestational age births [8].

Successful long-term weight loss is rarely achieved through conservative treatment alone, even though there are evidence of the moderate effectiveness of combination of psychological intervention, exercise and dietary strategies [9]. Bariatric surgery is the most effective therapy available today, with proven effects on long term weight loss, mortality [10, 11], morbidity [12, 13] and incidence of cancer in women [14].

Bariatric procedures alter the anatomy of the gastrointestinal tract with the purpose of either restricting caloric intake or reducing absorption of food nutrients. All surgical procedures entail risks of complications. In bariatric surgery these risks mainly comprises malabsorption and surgical complications related to leakage from anastomoses or internal herniation of small bowel. In recent years new endoscopic techniques have emerged, thus providing less invasive options for treatment of obesity [15, 16].

We evaluated a new device for treating obesity, the AspireAssist® Aspiration Therapy System (Aspire Bariatrics, King of Prussa, PA), which consists of an endoscopically placed gastrostomy tube and siphon assembly. AspireAssist® allows patients to aspirate gastric contents 20 min after meal consumption three times daily. Aspiration takes about 10 min to perform and removes approximately 30% of ingested calories.

A pilot study by Sullivan et al. [17] reported significantly greater loss of excess weight with Aspiration Therapy in combination with lifestyle intervention, mean 49.0 (SEM 7.7)%, than lifestyle intervention alone, mean 14.9 (SEM 12.2)%, after 1 year follow-up. The authors also concluded that aspiration therapy does not induce any adverse eating behaviors or change baseline depression scores. All patients were supplemented with potassium chloride (20 mmol orally daily) and started on omeprazole (20 mg orally daily) to inhibit gastric acid secretion.

Irrigation and aspiration of gastric content can possibly lead to chronic loss of hydrogen and chloride ions. The physiologic response is renal secretion of potassium ions and resorption of hydrogen ions, resulting in hypochloremic hypokalemic metabolic alkalosis [18, 19]. Loss of potassium ions cause hypokalemia and risk of cardiac arrhythmia.

We have previously reported preliminary results of treatment with 6 months follow-up [20]. The aim of the current study was to evaluate weight loss, safety and quality of life with AspireAssist® treatment for 1 to 2 years in obese subjects.

## Methods

### Study design

A single-center, observational, 1-year, prospective study was conducted at the Department of Surgery at Blekinge County Hospital in Karlskrona, Sweden. Power calculation for sample size was not performed due to lack of published reports of AspireAssist®. This study was designed as an initial exploratory study of safety and effectiveness. Subjects were consecutively recruited after advertisement in local media. Inclusion criteria were BMI ≥ 35.0 kg/m^2^ and age from 25 to 65 years. Exclusion criteria were myocardial infarction during the last 3 months, known malignancy, chronic liver or kidney disease, prior major surgery in the upper gastrointestinal tract, psychiatric disease including substance abuse, eating disorder, mental retardation or other intellectual disability. Participants had the option to continue therapy for an additional year.

### Aim

The aim of the current study was to evaluate weight loss, safety and quality of life with AspireAssist® treatment for 1 to 2 years in obese subjects.

### Subjects

Twenty-five obese subjects were enrolled between July and September 2012. All subjects underwent interview and medical examination. There were 23 women and two men. Median age was 48 years (range 33–65). Baseline comorbidities are presented in Table 1.

**Table 1:** Comorbidities under treatment at baseline and after 12 months aspiration therapy

Comorbidities under treatment Number of subjects	Baseline, all subjects *n* = 25	Baseline, per protocol *n* = 20	12 months, per protocol *n* = 20
Diabetes mellitus type 2 (total)	7	7	5
- Diet treatment	2	2	2
- Metformin	4	4	2
- Metformin + insulin	1	1	1
Hypertension (total)	9	8	7
- Diuretic	6	5	4
- ACE-inhibitor	5	5	4
- Beta receptor antagonist	3	3	3
Hyperlipidemia (total)	2	2	2
- Statin	2	2	2
Mood disorder (total)	9	6	6
- SSRI	8	6	6
- Tricyclic antidepressant	1	0	0
Gastroesophageal reflux disease	2	2	3
- Omeprazole	2	2	3

### Study protocol

The components in the AspireAssist® Aspiration Therapy System and the technique for endoscopic placement of the A-tube are described by Sullivan et al. [17]. Changes from the protocol used by Sullivan et al. include addition of preoperative diet, use of ultrasound, change in prophylactic antibiotics and elimination of prophylactic prescription of potassium and proton pump inhibitors.

#### Preoperative diet

Consistent with the pre-surgical regimen for bariatric surgery in Sweden, subjects were placed on a 4 week preoperative very low calorie diet (VLCD) (Slanka, Slanka Sverige AB, Sweden) providing approximately 680 kcal per day. Preoperative diet reduces liver size [21] and thereby, theoretically, lowers the risk of iatrogenic liver damage.

#### Placement of the A-tube

The gastrostomy tube (the AspireAssist® A-tube) was placed as a conventional pull percutaneous endoscopic gastrostomy (PEG) and was performed under conscious sedation (midazolam and ketobemidone). A standard pull PEG set (Boston Scientific Europe, France) was used. Careful evaluation of the optimal site for the placement of the A-tube was performed in the epigastric part of the abdomen with transillumination and finger indentation at endoscopy. Bedside ultrasound examination was performed to mark the border of the left liver lobe. All patients received a postoperative prescription of paracetamol and codeine.

#### Prophylactic antibiotics

Immediately after the procedure all patients received a single dose of sulfamethoxazole and trimethoprim orally [22].

#### Placement of skin-port

Approximately 14 days after A-tube placement, allowing the fistula to be formed, the tube was cut at the level of the skin and a low-profile valve (the AspireAssist® Skin-Port) was installed. The tube was subsequently shortened and the Skin-port readjusted following weight-loss.

#### Aspiration therapy

As reported by Sullivan et al. [17], subjects were instructed to aspirate 20 min after breakfast, lunch, and dinner. To prevent the tube from clogging when attempting to aspirate, patients were instructed to chew their food thoroughly. The AspireAssist® Connector opens the valve and enables irrigation with tap water and aspiration of gastric contents. The Connector counts down after each connection and is disabled after 115 cycles, preventing additional aspirations, and hence requires the subject to return to a healthcare professional to obtain a new Connector.

#### Cognitive behavioral therapy

Cognitive behavioral therapy (CBT) weight loss program with elements of psychoeducation and mindfulness was provided along with aspiration therapy by a CBT-certified therapist and psychologist. Two sessions were performed individually and six sessions were performed in group during the initial 3 months of aspiration therapy. In total each subject participated in eight sessions of CBT. In addition to CBT each subject had seven appointments with the project out-patient nurse for clinical control and cutting of external excess tube length.

#### Assessment of quality of life

Quality of life was assessed by EuroQoL 5-dimension (EQ-5D, http://www.euroqol.org) questionnaires and by a visual analogue scale (VAS) of EQ-5D at baseline and at 52-weeks.

#### Electrolyte and pharmacological considerations

The risk of slowly developing intracellular potassium deficiency regardless of normal potassium in blood samples motivated measurement of total potassium ion content in 24 h urine samples to detect an increase in potassium losses. Six subjects were on diuretics for blood pressure control and were supplemented with oral potassium chloride 20 mmol, daily. All subjects were instructed to administer any oral medications after aspiration, or 2 h before aspiration.

#### Monitoring

Subjects had regular contact with the out-patient nurse; initially four times during the first 3 months, thereafter every 3 months. Blood and urine samples were collected and weight registered every 3 months. Medical charts were reviewed, and subjects were inquired for adverse events, at each study visit. The Clavien-Dindo classification of surgical complications [23, 24] was used for grading of complications.

### Statistical analysis

The statistical analysis was performed using Stata version 14 (StataCorp LP, College Station, Texas, USA). Skewness/Kurtosis test was used for normality assessment. Parametric variables are presented as mean with standard deviation (SD), and compared by two sample student *t* test. Nonparametric variables are presented as median with inter quartile range (IQR), and compared with Wilcoxon matched-pairs signed-ranks test. A two-sided *p*-value of ≤0.050 was considered statistically significant. Data in Fig. 2 and Table 2 is presented both as “per protocol” (*n* = 25 at baseline, *n* = 20 at 12 months and *n* = 15 at 24 months) and as intension to treat (*n* = 25) with the last registered value carried forward.

BMI is calculated from body mass in kg divided with the square of patient length in meters. Percent excess weight loss is weight loss in percent exceeding BMI 25: ((baseline BMI - BMI at measure point)/(baseline BMI - 25)) *100.

## Results

### Compliance

**Fig. 1:**
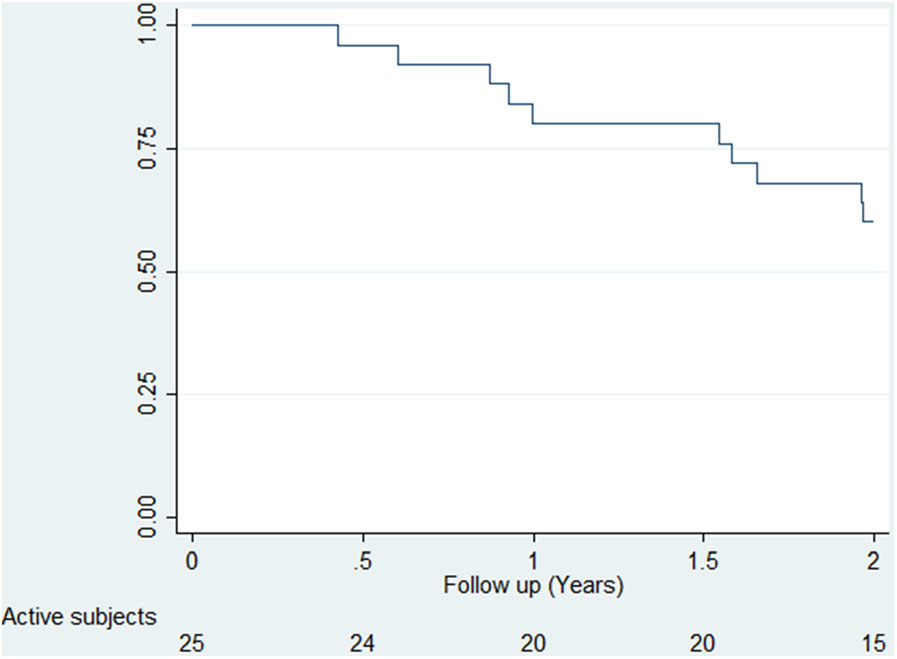
Subject participation during 2 years with aspiration therapy

Twenty of the subjects (80%, one man and 19 women) completed the intended 12-months of aspiration therapy (Fig. 1). Five subjects withdrew from the study prior to 1-year, primarily due to lack of motivation and unwillingness to comply with the regimen. Sixteen subjects experienced the Aspire method functioning and chosed to continue treatment for a second year. One of the 16 subjects withdrew before completion of the second year. In one case the proximal end of the A-tube had migrated from the fundus of the stomach down into the bulb of the duodenum and thereby constituting an outlet obstruction which was consistent with the vomiting and nausea reported by the patient. In all subjects that aborted the treatment, the A-tubes were removed in a secondary gastroscopy and the fistula closed spontaneously thereafter.

Frequency of aspirations was registered in a personal diary during the first year of treatment. Aspiration three times daily was reported for 76% of days for subjects in active treatment. A detailed analysis of the diary data was not performed, but a tendency was noted that weight loss correlated with frequency of aspirations. Frequency of aspirations was reduced after consistent weight loss.

### Weight

**Fig. 2:**
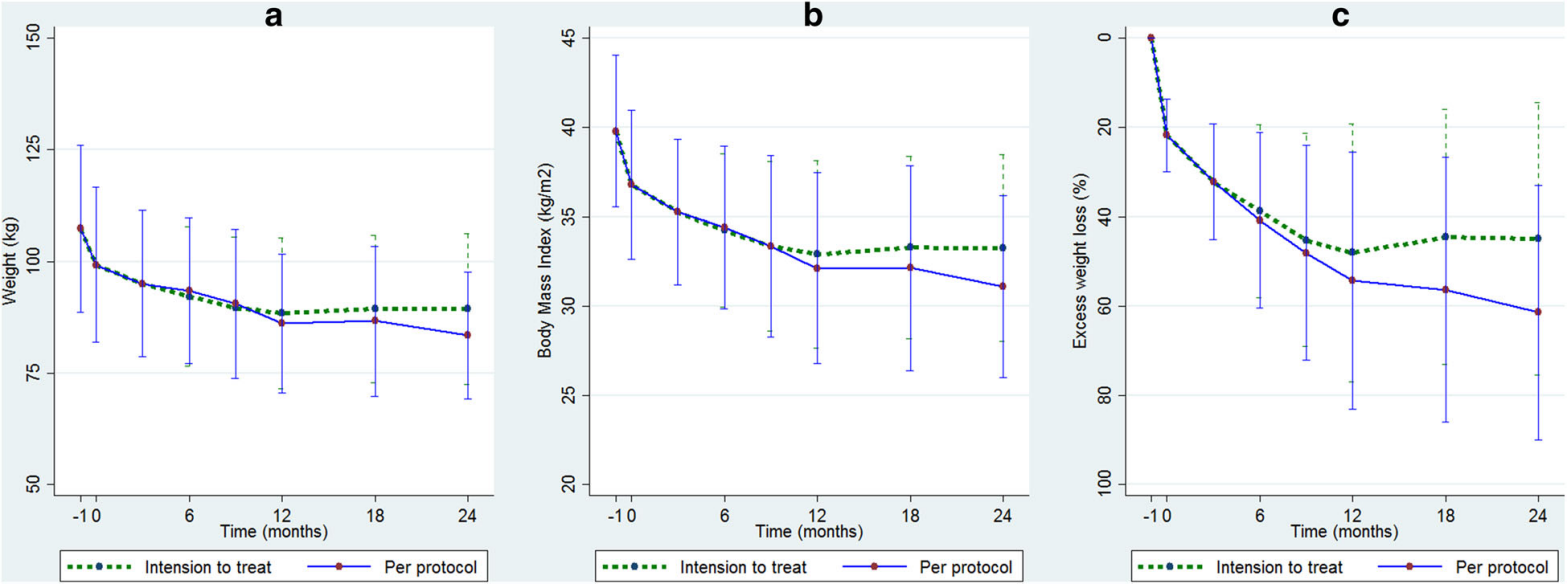
Weight loss during 24 months aspiration therapy. Baseline is “-1 month” followed by 4 weeks very low calorie diet before insertion of A-tube at “0 months”. Excess weight is weight exceeding BMI of 25. Data are presented as mean values with standard deviation. **a** Weight. **b** Body Mass Index. **c** Excess weight loss

Weight data is presented in Fig. 2 and Table 2. Mean baseline weight was 107.4 kg (SD 18.7) and mean baseline excess weight (defined as weight exceeding BMI 25) was 40.2 kg (SD 13.8). Mean excess weight loss per protocol was 54.4% (SD 28.8) at 12 months and 61.5% (SD 28.5) at 24 months.

**Table 2:** Results of 24 months aspiration therapy on weight presented as per protocol and as intention to treat with last value carried forward

Weight data Mean (SD)	Baseline	12 months	*p*	24 months	*p*
Per protocol	*n* = 25	*n* = 20		*n* = 15	
Weight (kg)	107.4 (18.7)	86.1 (15.5)	<0.01	83.4 (14.2)	<0.01
BMI (kg/m^2^)	39.8 (4.3)	32.1 (5.4)	<0.01	31.1 (5.1)	<0.01
Excess weight (kg)	40.2 (13.8)	19.0 (14.8)	<0.01	16.1 (13.7)	<0.01
Excess weight loss (%)	N.A.	54.4 (28.8)	N.A.	61.5 (28.5)	N.A.
Intention to treat (Last value carried forward)	*n* = 25	*n* = 25		*n* = 25	
Weight (kg)	107.4 (18.7)	88.4 (16.9)	<0.01	89.3 (16.9)	<0.01
BMI (kg/m^2^)	39.8 (4.3)	32.9 (5.3)	<0.01	33.2 (5.2)	<0.01
Excess weight (kg)	40.2 (13.8)	21.2 (14.9)	<0.01	22.1 (14.8)	<0.01
Excess weight loss (%)	N.A.	44.5 (28.8)	N.A.	44.9 (30.5)	N.A.

### Procedure safety - electrolytes

**Fig. 3:**
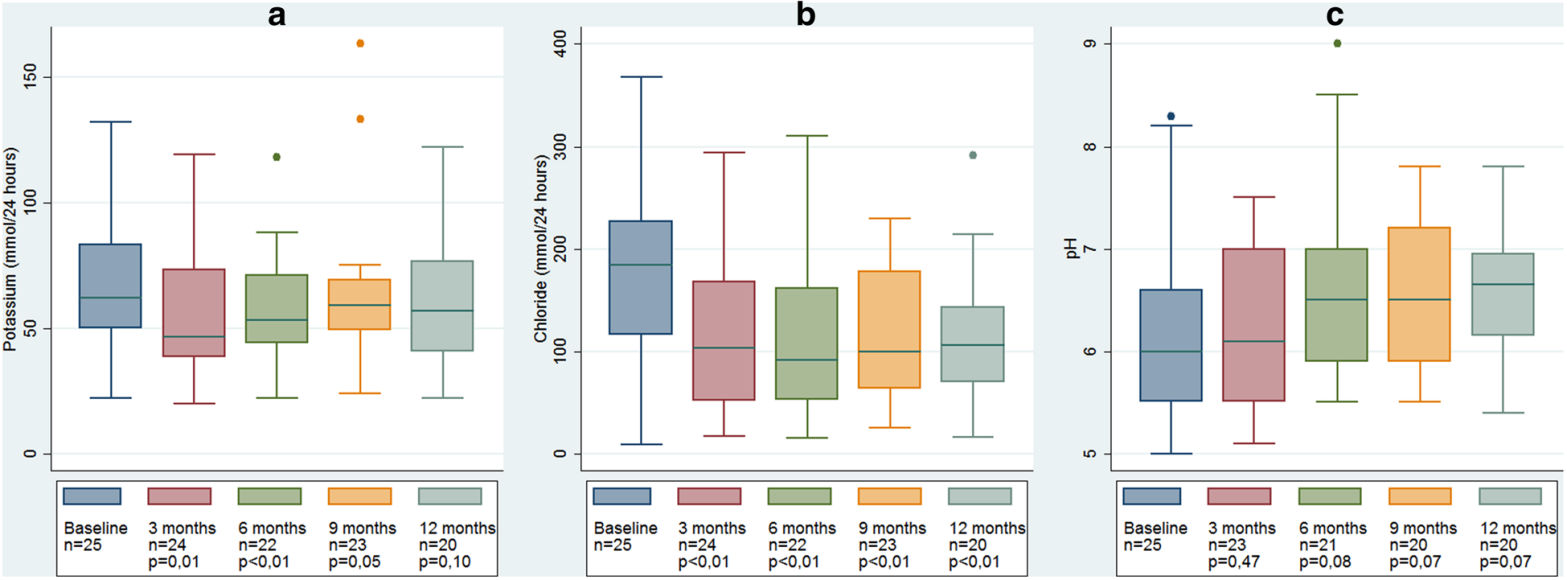
Change in urinary pH and electrolyte secretion during 12 months aspiration therapy. **a** Potassium secretion during 24 h. **b** Chloride secretion during 24 h. **c** pH

Changes in electrolyte values during aspiration therapy are presented in Table 3. There were neither significant differences in serum potassium concentrations nor any significant difference in total urinary secretion of potassium in 24 h samples, as presented in Fig. 3. Urine samples showed a trend of increasing pH in the urine, indicating alkalization of urine, but the difference was not statistically significant. Change of the urine pH value is illustrated in Fig. 3.

**Table 3:** Results of 12 months aspiration therapy on quality of life, blood and urine values and HbA1c level (the latter for diabetic subjects only)

Quality of life and biochemical data	Baseline *n* = 20	12 months *n* = 20	*p*
Quality of life			
Visual Analogue Scale*	63 (15)	83 (14)	<0.01
EuroQoL-5D*	0.73 (0.25)	0.88 (0.13)	<0.01
Blood samples			
Potassium (mmol/l)	4.4 (4.2–4.5)	4.3 (4.0–4.5)	0.57
Sodium (mmol/l)	140 (139–141.5)	141.5 (140–142)	0.02
Creatinine (umol/l)	62.5 (53–67)	68 (58.5–73.5)	<0.01
Urine samples			
24 h potassium (mmol)	66 (54–87)	57 (40.5–76.5)	0.10
24 h sodium (mmol)	203 (122–267)	147.5 (110.5–188.5)	0.02
24 h chloride (mmol)	188 (141.5–246.5)	106 (70–143)	<0.01
Osmolality (mmol/kg)	482 (377–681)	394 (335–571)	0.30
pH	6.0 (5.5–6.7)	6.7 (6.2–7.0)	0.02
Subjects with diabetes mellitus	Baseline *n* = 7	12 months *n* = 7	*p*
Blood samples			
HbA1c (mmol/mol)	47 (43–66)	42 (36–64)	0.03

The five subjects who aborted treatment are excluded (presentation as per protocol at 1 year). Data are presented as median (IQR) or mean (SD), the latter indicated by *

Among the seven diabetic patients there was a significant reduction in glycosylated hemoglobin type A1c (HbA1c) level from median 47 (IQR 43–66) mmol/mol to median 42 (IQR 36–64) mmol/mol, *p* = 0.03. Some of the subjects were able to reduce or withdraw their oral medications for diabetes, as presented in Table 1.

### Procedure safety - complications

During the first postoperative week 13 subjects (52%) reported moderate pain (relieved by analgesics) and three subjects (9%) reported severe pain. Two were admitted to hospital and underwent computed tomography due to suspicion of leakage or tube displacement. One subject had no signs of leakage or displacement, and improved promptly under observation. The other had a small intraabdominal leakage at the gastrostomy site, a small cavity with fluid was drained with ultrasound guidance and subsequent culture was negative.

Three subjects reported additional adverse events during the first 30 days postoperative. All of them were stoma site related problems (one conservatively treated skin irritation, one required antibiotics orally for bacterial infection, and one had a small skin breakdown around the stoma that resolved with conservative treatment).

In the subsequent 11 months one subject had a bacterial infection at the stoma, treated with antibiotics orally. Two subjects presented in the emergency department with gallstones, one who also had pancreatitis. Both underwent laparoscopic cholecystectomy.

According to the Clavien-Dindo grading system there were one grade III-a, two grade II and three grade I complications within 30 days and one more grade II complication during the consequent 11 months. Postoperative pain was not classified as a complication if it did not cause hospitalization. Gallstone diseases presented early at a time where weight-loss was modest and were hence not regarded as direct complications to the aspiration therapy.

### Quality of life

**Fig. 4:**
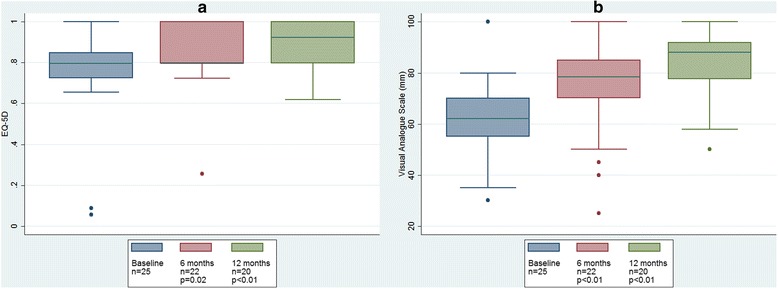
Change in quality of life during 12 months aspiration therapy. **a** EuroQoL 5-dimensions (EQ-5D). **b** Visual analogue scale

Subjects reported that quality of life, as measured with EQ-5D and VAS, significantly increased during treatment. This is presented in Table 3 and Fig. 4.

## Discussion

The aim of this study was to evaluate weight reduction and safety of aspiration therapy with AspireAssist™. Twenty of our 25 subjects completed the initial 1 year treatment. These 20 subjects lost mean 54% of their excess weight. At 2 years, 15 subjects had lost mean 61% of their excess weight. This weight loss surpassed our expectation and is nearly at the level of gastric bypass procedure and other major abdominal surgery for obesity. The subjects reported improved quality of life during treatment. There was neither mortality nor any event more severe than grade III-a according to Clavien-Dindo grading system.

The hypothesis of a possible compensatory increase in potassium secretion into urine, which could lead to chronic hypokalemia and subsequent risk of cardiac arrhythmia, was examined and rejected. We therefore conclude that prophylactic administration of potassium or proton pump inhibitors is unnecessary, if the patient does not use any potassium lowering medication (as for example diuretics). We also observed that aspiration therapy caused alkalization of urine and a reduced urinary secretion of chloride and sodium. We interpret this as a compensatory response due to loss of some hydrochloric acid.

Drainage of stomach content is only possible if the patient eats slowly and chews thoroughly. The patient gets a direct feedback regarding how well he or she chewed when attempting aspiration. Many subjects reported that the treatment has changed their eating habits, with slower eating and smaller meals. This change may in itself lead to a reduction in caloric intake. The previous study by Sullivan and his colleges [17] indicates that the treatment does not lead to increase in caloric intake.

Aspiration therapy requires patient cooperation, making compliance integral to success. Treatment can be intensified if there is indication of relapse with weight gain. Today we have no means of predicting compliance, but orderly persons with a capacity to follow daily routines seem to have a higher probability of success. In our study with 25 volunteers the drop out frequency was 20% the first study year, but 64% wanted to continue treatment for a second year (Fig. 4). The participants in our study reported an expected improvement in quality of life, corresponding to weight loss, despite the presence of a gastrostomy and the need to perform drainage of stomach contents three times daily.

Bariatric surgery is currently regarded as a golden standard for obesity treatment. Aspiration therapy provides a reversible, easily performed outpatient procedure that does not entail the risks associated with major abdominal surgery. AspireAssist® does not alter the anatomy of the gastrointestinal tract. In our experience so far, the AspireAssist® gastrostomy tube placement has not complicated subsequent laparoscopic bariatric surgery. One subject in our study underwent a gastric bypass procedure without any problems related to the prior gastrostomy stoma.

Limitation of this study is the combination of aspiration therapy and CBT without any control group. This study only encompasses treatment during 1 to 2 years. Long term patency is still unknown. It is our belief that once the desired weight goal is achieved many, if not most, patients will need to continue aspiration therapy, albeit possibly at a reduced frequency, to maintain weight stability. In order to determine this we have started a prospective study in which we will follow 50 patients with AspireAssist® and 50 patients with laparoscopic gastric bypass procedure for 5 years.

## Conclusions

Aspiration therapy is a safe method that allows the motivated obese patient to reduce his or her excess weight by half, thus improving quality of life, in 1 year’s time. Long term results remain still to be investigated.

## Abbreviations

BMI: Body mass index
CBT: Cognitive behavioral therapy
EQ-5D: EuroQoL 5-dimension
IQR: Inter quartile range
PEG: Percutaneous endoscopic gastrostomy
SD: Standard deviation
SEM: Standard error of the mean
VAS: Visual analogue scale
VLCD: Very low calorie diet
